# Evaluation of nine candidate genes in patients with normal tension glaucoma: a case control study

**DOI:** 10.1186/1471-2350-10-91

**Published:** 2009-09-15

**Authors:** Christiane Wolf, Eugen Gramer, Bertram Müller-Myhsok, Francesca Pasutto, Eva Reinthal, Bernd Wissinger, Nicole Weisschuh

**Affiliations:** 1 Centre for Ophthalmology, Institute for Ophthalmic Research, Molecular Genetics Laboratory, Tuebingen, Germany; 2 University Eye Hospital, Julius-Maximilians-University, Wuerzburg, Germany; 3 Max Planck Institute of Psychiatry, Munich, Germany; 4 Institute of Human Genetics, Friedrich-Alexander-University Erlangen-Nuremberg, Erlangen, Germany; 5 Centre for Ophthalmology, University Eye Hospital, Tuebingen, Germany

## Abstract

**Background:**

Normal tension glaucoma is a major subtype of glaucoma, associated with intraocular pressures that are within the statistically normal range of the population. Monogenic forms following classical inheritance patterns are rare in this glaucoma subtype. Instead, multigenic inheritance is proposed for the majority of cases. The present study tested common sequence variants in candidate genes for association with normal tension glaucoma in the German population.

**Methods:**

Ninety-eight SNPs were selected to tag the common genetic variation in nine genes, namely OPTN (optineurin), RDX (radixin), SNX16 (sorting nexin 16), OPA1 (optic atrophy 1), MFN1 (mitofusin 1), MFN2 (mitofusin 2), PARL (presenilin associated, rhomboid-like), SOD2 (superoxide dismutase 2, mitochondrial) and CYP1B1 (cytochrome P450, family 1, subfamily B, polypeptide 1). These SNPs were genotyped in 285 cases and 282 fully evaluated matched controls. Statistical analyses comprised single polymorphism association as well as haplogroup based association testing.

**Results:**

Results suggested that genetic variation in five of the candidate genes (RDX, SNX16, OPA1, SOD2 and CYP1B1) is unlikely to confer major risk to develop normal tension glaucoma in the German population. In contrast, we observed a trend towards association of single SNPs in OPTN, MFN1, MFN2 and PARL. The SNPs of OPTN, MFN2 and PARL were further analysed by multimarker haplotype-based association testing. We identified a risk haplotype being more frequent in patients and a vice versa situation for the complementary protective haplotype in each of the three genes.

**Conclusion:**

Common variants of OPTN, PARL, MFN1 and MFN2 should be analysed in other cohorts to confirm their involvement in normal tension glaucoma.

## Background

The term glaucoma describes a heterogeneous group of optic neuropathies. Yet progressive loss of retinal ganglion cells (RGCs) and optic nerve axons, corresponding visual field loss and characteristic optic nerve head cupping is common in all forms of glaucoma. The three major risk factors for the development of glaucoma are elevated intraocular pressure (IOP), advanced age and a positive family history. It is estimated, that by 2010, 60 million people will suffer from glaucoma and that the disease will be the second leading cause of blindness worldwide [1].

Primary open angle glaucoma (POAG) is the most frequent of all glaucoma types. In approximately one third of all Caucasian POAG patients the IOP is within the average range of 10-21 mmHg. Nonetheless such patients with normal tension glaucoma (NTG) show typical pathological cupping of the optic nerve as well as characteristic visual field defects [2,3].

First degree relatives of glaucoma patients have an elevated risk to develop glaucoma themselves, indicating that genetic risk factors are involved in the etiology of POAG.

Though at least 14 loci have been linked to POAG (GLC1A-GLC1N), only three causative genes have been identified to date: myocilin (MYOC/GLC1A), optineurin (OPTN/GLC1E), and WD repeat domain 36 (WDR36/GLC1G). Altogether, mutations in these three genes account for less than 10% of POAG cases [4]. In addition, association studies have implicated more than 20 other genes in POAG [5]. Most of these genes were investigated only in single studies whereas the OPA1 gene, which is linked to autosomal dominant optic atrophy, has been subject of multiple studies. However, results were contradictory [6-11].

Taken together, true monogenic forms following classical inheritance patterns are rare in POAG. It is therefore generally accepted that the majority of cases are most likely to evolve from multigenic inheritance, i.e. genes interacting synergistically to cause the condition [12].

Therefore, association studies may be suitable to unravel the genetic etiology of multigenic human diseases like glaucoma. The recent identification and replication of the LOXL1 gene as a risk factor for pseudoexfoliation glaucoma [13] clearly demonstrates the power of association studies in glaucoma research.

In the present study, we performed a case-control study to identify genetic risk factors for the development of NTG. A tagging single nucleotide polymorphism (tagSNP) approach based on linkage disequilibrium (LD) blocks was used to determine the frequency of common sequence variants in nine candidate genes. The genes we analysed were OPTN (optineurin), RDX (radixin), SNX16 (sorting nexin 16), OPA1 (optic atrophy 1), MFN1 (mitofusin 1), MFN2 (mitofusin 2), PARL (presenilin associated, rhomboid-like), SOD2 (superoxide dismutase 2, mitochondrial) and CYP1B1 (cytochrome P450, family 1, subfamily B, polypeptide 1).

Mutations in the OPTN gene were initially reported in 16.7% of families with hereditary POAG and NTG [14]. Follow-up studies in unselected cohorts however indicated that coding OPTN sequence variants are only a rare cause of POAG or NTG in different populations including our own German NTG cohort [15-18]. This however does not rule out that other common gene variations in OPTN not yet investigated (e. g. in introns or noncoding flanking regions) may show up as risk associated variants in a case control study. In addition to OPTN we also considered RDX and SNX16, since both genes are linked with OPTN expression [19].

Mutations in the OPA1 gene are a frequent cause of autosomal dominant optic atrophy [20]. In addition, common sequence variants in the OPA1 gene have been associated with POAG or specifically NTG, though the findings could not be replicated in all populations [6-11]. In the present study, we extended the analysis by genotyping 16 common variants in the genomic region of the OPA1 gene.

Two genes that encode functional interaction partners of the OPA1 protein were also included in our study: MFN1, which acts in concert with OPA1 in the mitochondrial fission and fusion process [21], and PARL, which encodes a processing protease for OPA1 [22-24]. We also analysed the MFN2 gene, as the MFN2 protein shows functional overlap with OPA1 [25].

The SOD2 gene encodes the Mn_2 _dependent superoxide dismutase that acts as a primary scavenger of reactive oxygen species in mitochondria. Oxidative stress is proposed to be involved in the etiology of glaucoma [26] and increased expression of the SOD2 gene was observed in the aqueous humor of glaucoma patients [27].

Mutations in the CYP1B1 gene cause an autosomal recessive form of primary congenital glaucoma [28] and two common sequence variants (Val432Leu and Asn453Ser) in the CYP1B1 gene are proposed to act as a risk factor for the development of POAG [29] and to influence clinical features like optic disk cupping and visual field deterioration in POAG patients [30]. Moreover, a very recent study showed that heterozygous CYP1B1 mutations with absent or reduced relative enzymatic activity are associated with POAG [31].

The goal of the present study was to determine whether common sequence variants in nine functionally defined candidate genes influence the risk to develop NTG in a large homogeneous patient cohort and would thus be worthy of more detailed examination.

## Methods

### Study samples

285 unrelated German patients with a diagnosis of NTG were recruited at the University Eye Hospital Wuerzburg, Germany. All patients were traced back to the third generation to reconfirm ethnicity. NTG was defined by (1) the presence of typical glaucomatous optic neuropathy with corresponding visual field loss, (2) open drainage angles on gonioscopy, (3) absence of a secondary cause for glaucomatous optic neuropathy, such as previously elevated IOP after trauma, a period of steroid administration or uveitis, and (4) IOP measures of untreated NTG continuously 21 mmHg or lower on repeated diurnal testing (five readings between 8:00 AM and 6:00 PM). The median of readings was required to be 21 mm Hg or less, with no reading above 24 mm Hg and no more than one reading of 23 or 24 mm Hg. IOP readings were correlated with corneal thickness. Patients did not have evidence of high myopia or congenital ocular abnormality and had no other cause than glaucoma for disc changes and visual field loss. Disc size and parameters were evaluated by confocal examination (Heidelberg Retina Tomograph). To minimize interobserver variability, more than 95% of patients were examined by the same ophthalmologist. All patients had a long term follow-up to ensure diagnosis of NTG with a maximum of certainty. In large part, NTG patients underwent a neurological examination to exclude an intracerebral expansion. Sonography was used to rule out aortic stenosis.

The control cohort comprised 282 unrelated German individuals, matched with the patient cohort for age, gender distribution and geographical origin. Ophthalmic examination was performed in all control subjects, including IOP measurement and ophthalmoscopy of the optic disc, to ascertain an IOP in the normal range (<21 mmHG) and the absence of glaucomatous disc change. Controls had no family history of glaucoma.

This study adhered to the tenets of the Declaration of Helsinki. Written informed consent was obtained from all subjects and the study was approved by the ethics committees of the University of Tuebingen, the University of Wuerzburg and the University of Erlangen-Nuremberg.

The average age of the patient group was 64.7 ± 13.9 years (controls: 66.9 ± 13.1 years) with a median of 67 years (controls: 68 years). Thirty-four percent of the NTG patients were male and 66% were female, while 41% of the controls were male and 59% were female.

### Isolation of DNA, SNP selection and genotyping

DNA was extracted from peripheral blood lymphocytes using the Magnetic Separation Module I from Chemagen using DNA chemistry (Chemagic DNA Blood Kit Special; Chemagen AG, Baesweiler, Germany).

Nine genes were investigated in this study, namely OPTN, RDX, SNX16, OPA1, MFN1, MFN2, PARL, SOD2 and CYP1B1.

Linkage disequilibrium (LD) blocks were defined using the algorithm by Gabriel and coworkers [32] implemented in Haploview [33] (version 4.1) on the HapMap31 CEPH (Utah residents with ancestry from northern and western Europe) data. LD blocks covered the chromosomal regions of these genes extending to the neighbouring genes. TagSNPs capturing LD blocks were selected to define haplotypes having a frequency of at least 0.1 and a minor allele frequency (MAF) of 0.1 or higher. Additional non-tagging SNPs were genotyped in the OPA1 gene (rs166850 and rs10451941) and in the SOD2 gene (rs4880), respectively. For the CYP1B1 gene, only two coding SNPs were selected for genotyping (rs1056836 and rs1800440).

The number of SNPs genotyped in each gene is shown in Table 1. All SNPs were genotyped with TaqMan assays (Applied Biosystems, Foster City, USA). Assay IDs and orientation of design strands are given in Additional file 1. Reactions were prepared according to the manufacturer's instructions and run on a sequence detection system (ABI 7500) using standard protocols. Sequence data was analysed using Sequence Detection Software version 1.2.3.

**Table 1:** Chromosomal regions of genotyped SNPs

**Gene**	**CHR**	**included chromosomal region (bp)**	**number of genotyped SNPs**
*CYP1B1*	2	64	2
*MFN1*	3	60496	5
*MFN2*	1	55573	4
*OPA1*	3	208893	16
*OPTN*	10	106314	29
*PARL*	3	109822	7
*RDX*	11	151216	14
*SNX16*	8	204923	11
*SOD2*	6	123647	10

CHR, chromosome; bp, basepairs

For quality-control reasons, 28 of the 98 TaqMan assays used in this study were selected randomly to confirm standard individual genotypes. Altogether, 1294 genotyping reactions were performed on 58 CEPH individuals. These CEPH individuals comprised 48 individuals that are identical with the triplets used in the HapMap [34] project and 10 additional members of one of the families. Genotyping results were checked for pedigree consistency and a cross comparison with HapMap data revealed a 100% concordance.

### Statistical analysis

All statistical analyses were performed using PLINK [35] (version 1.05). The minimum genotyping rate for SNPs and individuals to be included in the statistical analyses was set to 90%.

Hardy-Weinberg equilibrium was assessed by using an exact test. Case-control association testing of single SNPs was performed by logistic regression. Multiple comparisons of SNPs covering all investigated genes were corrected using the Bonferroni method.

Interaction of SNPs was analysed by an SNP × SNP epistasis test with subsequent correction for multiple comparisons by the Bonferroni method. Haplotype-based case control association testing was assessed as implemented in PLINK.

## Results

Ninety-eight SNPs were selected to tag the common genetic variation in OPTN, RDX, SNX16, OPA1, MFN1, MFN2, PARL, SOD2 and CYP1B1.

One of these (RDX: rs1676536) was excluded from further analysis due to a low genotyping rate. The remaining panel of SNPs was genotyped in a case-control study in 285 patients with NTG and 282 healthy controls of German origin. The average number of individuals remaining for analysis was 554.31 ± 2.5 (median 555) per SNP and the average genotyping rate in these individuals was 0.995 ± 0.007 (median 0.997). The size of the chromosomal regions and the number of SNPs genotyped in each gene are shown in Table 1. For all SNPs, genotype distributions in patients and controls were consistent with Hardy-Weinberg equilibrium.

We found no evidence of association between NTG and RDX, SNX16, OPA1, SOD2 and CYP1B1. Entire allele frequencies and detailed results of the logistic regression of single SNP association testing are provided in Additional file 1.

We identified a nominally significant association of NTG with four SNPs in OPTN (rs10796021, rs3829923, rs7921853 and rs765884), three almost consecutive SNPs in the MFN2 gene (rs873458, rs2295281 and rs11588779), two close neighboring SNPs in PARL (rs1000002 and rs1402003) and one SNP in the MFN1 gene (rs2111534) (Table 2). Genewise case-control comparison of multimarker haplotypes derived from the SNPs in OPTN, MFN2 and PARL revealed two complementary common haplotypes for each gene. The differences of these haplotype frequencies between cases and controls were nominally significant (Table 3). In PARL, a very rare haplotype showed a nominal association with NTG (Table 3).

**Table 2:** Allele frequencies and summary statistics for significant SNPs before adjustment for multiple testing

**Gene**^1^	**SNP**	**MA**	**MAF patients**	**MAF controls**	**OR**	**L95**	**U95**	**p**
*OPTN*	rs10796021	C	0.471	0.405	1.316	1.022	1.695	0.033
*OPTN*	rs3829923	T	0.375	0.444	0.768	0.602	0.980	0.034
*OPTN*	rs7921853	T	0.498	0.415	1.356	1.069	1.721	0.012
*OPTN*	rs765884	C	0.315	0.224	1.558	1.179	2.059	1.838 × 10^-3^
*MFN1*	rs2111534	A	0.384	0.450	0,748	0.583	0.960	0.023
*MFN2*	rs873458	G	0.496	0.432	1.292	1.019	1.639	0.035
*MFN2*	rs2295281	C	0.493	0.423	1.327	1.044	1.687	0.021
*MFN2*	rs11588779	T	0.221	0.282	0.721	0.547	0.950	0.020
*PARL*	rs1000002	C	0.539	0.437	1.478	1.091	2.002	0.012
*PARL*	rs1402003	G	0.464	0.368	1.471	1.079	2.006	0.015

^1^Refers to the gene for which LD blocks were calculated. MA, minor allele; MAF, minor allele frequency; OR, odds ratio; L95, confidence interval 95% lower bound; U95, confidence interval 95% upper bound

**Table 3:** Summary statistics on haplotype-based association

**Gene**	**SNP identifiers and omnibus association**	**Haplotype**	**F patients**	**F controls**	**χ^2^**	**p**
*OPTN*	rs10796021|rs3829923|rs7921853|rs765884	TTGT	0.257	0.327	5.905	0.015
	p_omnibus _= 0.014	CCGT	0.125	0.142	0.590	0.443
	χ^2 ^_omnibus _= 23.67	TCTT	0.129	0.119	0.243	0.622
		CCTC	0.187	0.093	18.170	2.021 × 10^-5^
		TCTC	0.062	0.073	0.484	0.487
		CCTT	0.072	0.070	0.019	0.890
		CTGT	0.048	0.056	0.293	0.588
		TTTT	0.036	0.040	0.083	0.773
		TCGT	0.023	0.031	0.728	0.394
		CCGC	0.029	0.029	0.003	0.959
		CTTC	0.014	0.013	0.023	0.880
		TTGC	0.018	0.009	1.611	0.204
*MFN2*	rs873458|rs2295281|rs11588779	ATC	0.410	0.420	0.1083	0.742
	p_omnibus _= 0.018	GCC	0.359	0.286	6.541	0.011
	χ^2 ^_omnibus _= 11.97	ATT	0.091	0.147	8.334	3.891 × 10^-3^
		GCT	0.131	0.135	0.039	0.843
		GTC	0.009	0.011	0.099	0.753
*PARL*	rs1000002|rs1402003	TA	0.461	0.540	4.049	0.044
	p_omnibus _= 7.619 × 10^-4^	CG	0.464	0.344	9.758	1.786 × 10^-3^
	χ^2 ^_omnibus _= 16.84	CA	0.075	0.093	0.7123	0.399
		TG	0	0.023	8.480	3.590 × 10^-3^

F, frequency

For correcting data for multiple comparisons we also considered our previously performed association study with the same cohorts [36]. Since a p value of less than 4.808 × 10^-4 ^was considered to be significant applying Bonferroni adjustment over the whole study, no significance of any single SNP association in any gene was further observed after correction for multiple comparisons. The epistasis test for interaction of SNPs revealed no significant result after correction for multiple testing (data not shown).

## Discussion

There is a growing number of single gene or single variant based association studies in glaucoma. Our study based on the genotyping of tagSNPs to maximize genetic coverage of nine candidate genes (OPTN, RDX, SNX16, OPA1, MFN1, MFN2, PARL, SOD2 and CYP1B1). In total, we investigated 98 SNPs in 285 NTG patients and 282 controls and identified a trend towards association of NTG with common sequence variants of OPTN, MFN1, MFN2 and PARL.

No association study with RDX, SNX16 and SOD2 has been performed in glaucoma patients before. We hypothesized that genetic differences in these genes could confer risk to NTG since data from our previous work as well as from the literature point to a putative involvement of these genes in glaucoma related pathogenesis. However, our approach failed to find any association of NTG with any of the 35 examined SNPs in these genes suggesting that genetic variants at these loci are not of high relevance in the development of NTG in German patients.

In patients with NTG, the appearance of the optic nerve head is similar to that in autosomal dominant optic atrophy (ADOA) [37]. These similarities make OPA1, the major gene involved in ADOA [20], an excellent candidate gene for glaucoma, particularly NTG. Several studies showed an association of two intron 8 polymorphisms (rs166850 and rs10451941) in the OPA1 gene with NTG in Caucasians [6,7]. These studies were based on relatively small sample sizes comprising 61 to 83 patients. A more comprehensive study with 279 Caucasian POAG patients reported a lack of association similar to our findings [11]. As we did not only genotype SNPs rs166850 and rs10451941 but 14 additional SNPs within a 209 kb region encompassing the OPA1 gene, our results are more conclusive since we cover the genetic population variability of the entire OPA1 gene. Considering patient number and cohort homogeneity as well as the size of the analysed OPA1 region, our study is the most comprehensive one on this matter to date.

Mutations in the CYP1B1 gene are a frequent cause of primary congenital glaucoma. In addition, two common coding polymorphisms in this gene might contribute to the development of POAG. The Leu432Val variant (rs1056836) is associated with POAG in Indian patients [29] and the Asn453Ser (rs1800440) variant that is not directly associated with disease but with clinical features like optic disk cupping and visual field deterioration in French POAG patients [30]. We were not able to indentify an association of either of these two SNPs with NTG in our cohort. These conflicting findings may result from phenotypic differences (e.g. IOP level) between NTG and POAG. Inconsistent results may also be due to the large variability of CYP1B1 allele frequency distribution among different populations or the modest proportion of controls among the total number of samples in the previous studies [29,30].

The association of OPTN, PARL, MFN1 and MFN2 common sequence variants we observed justifies a more extensive analysis. The single SNP associations observed before adjustment for multiple comparisons are supported by multimarker haplotype-based associations that we found for OPTN, MFN2 and PARL. In each of the three genes, we identified a nominally significant risk haplotype being more frequent in patients and a vice versa situation for the complementary protective haplotype, both being present at common frequencies in either cohort. The relative risk for homozygous individuals of the OPTN risk haplotype which showed the strongest of all associations is 8.905 (95% CI: 1.477-54.313).

OPTN is one of three causative genes that have been linked to POAG [14]. Nonetheless, more than 25 studies dealing with the impact of OPTN coding and flanking intronic variants on glaucoma show that disease-causing sequence alterations are rare in POAG/NTG patients and families. However, the OPTN gene may be a risk factor in multigenic inheritance as synergistic effects with the two other POAG genes myocilin and WD repeat domain 36 have been suggested [38-40]. Yet, the mechanistic involvement of OPTN in glaucoma pathogenesis remains elusive. No case-control association study based on common gene variants has been performed for the complete chromosomal region of OPTN to date. Our study implicates that apart from OPTN mutations observed in monogenic inheritance of glaucoma also OPTN common allelic variants may influence an individual's susceptibility for developing NTG in a multigenic inheritance pattern.

MFN1 and MFN2 are nuclear encoded mitochondrial membrane proteins that interact with each other to facilitate mitochondrial targeting. MFN2 and OPA1 act in concert and conduct complementary functions in maintaining balance of mitochondrial fusion and fission events in the highly dynamic mitochondrial network architecture processes [25,41,42]. Likewise, PARL is a nuclear encoded mitochondrial protein and has been suggested to participate in OPA1 processing and apoptotic processes by regulation of cytochrome c release via OPA1-dependent cristae remodeling [22,43].

A possible involvement of mitochondrial function in mechanisms of glaucoma development has been indicated before [44-46]. According to the common disease-common variant hypothesis, multiple variants may each contribute a small additive or multiplicative effect to the disease phenotype. As mentioned above, the nuclear encoded mitochondrial genes OPA1, MFN1, MFN2 and PARL share functional overlap and/or are involved in joint mitochondrial pathways. However, we were not able to detect any significant interaction based on a SNP × SNP epistasis test between any of our nine potential candidate genes.

Most of the SNPs we analysed are located in intronic or intergenic regions, but may be in LD with functional coding or regulatory sequence variants. Considering our recruitment policy, we expect a highly homogeneous cohort as we applied strict diagnostic criteria. In addition, the ethnicity of all of our patients was traced back to the third generation. Therefore, we expect only small population admixture when comparing our cohort with such comprising a more genetically heterogeneous background (like for example the Unites States). As the homogeneity of a population corresponds with the persistence of LD between a genetic marker and a disease gene, the power to detect an association is higher in homogeneous populations. The homogeneity of our samples therefore may compensate in part for the small number of individuals. However, we are aware of the fact that our study is not well-powered for statistical testing. A retrospective power calculation based upon an OR of 1.558 (for SNP rs765884 which showed the strongest association) indicated that this study was sufficiently powered (91,8%; α = 0.05) to detect an effect of this magnitude or greater before correction for multiple comparisons. After Bonferroni correction, the power was considerably reduced (24,3%; α = 0.05).

## Conclusion

The objective of this study was to investigate association of several genes that have not been subject to an association study with glaucoma cases so far but might be functionally linked to glaucoma pathogenesis (RDX, SNX16, MFN1, MFN2, PARL, SOD2). In addition, we analysed genes that have been previously identified as candidate genes in glaucoma (OPTN, OPA1, CYP1B1). Whereas we could not detect any association of NTG with RDX, SNX16, OPA1, SOD2 and CYP1B1, our study revealed a trend towards association of NTG with common sequence variants of OPTN, MFN1, MFN2 and PARL, the latter three ones being involved in mitochondrial function.

Although our sample sizes are among the largest investigated in NTG so far, it is important to point out that the power of our study is still relatively limited. As we cannot fully exclude type one or type two errors, it is essential to replicate our findings in independent cohorts.

## Competing interests

The authors declare that they have no competing interests.

## Authors' contributions

CW carried out the genotyping, performed the statistical analysis, drafted the manuscript and participated in the design of the study. EG performed the clinical examination of all patients and participated in the coordination of the study. BM-M participated in the statistical analysis. FP and ER participated in the recruitment of control subjects. BW participated in study design and helped drafting the manuscript. NW conceived of the study, and participated in its design and coordination and helped to draft the manuscript. All authors read and approved the final manuscript.

## Pre-publication history

The pre-publication history for this paper can be accessed here:



## Supplementary Material

Additional file 1 **Supplementary table 1**. Allele frequencies and summary statistics for all SNPs. Click here for file
